# Freiburg Neuropathology Case Conference: Gait Ataxia, Segmental Hypoesthesia, and Combined Incontinence in a 57-Years-Old Patient

**DOI:** 10.1007/s00062-025-01528-1

**Published:** 2025-05-13

**Authors:** L. Miarka, U. Taschner, R. Rölz, M. Prinz, H. Urbach, D. Erny, C. A. Taschner

**Affiliations:** 1 Department of Neuropathology, Medical Centre, University of Freiburg, Freiburg, Germany; 2 Department of Neuroradiology, Medical Centre, University of Freiburg, Breisacherstraße 64, 79106 Freiburg, Germany; 3 Department of Neurosurgery Medical Centre, University of Freiburg, Freiburg, Germany; 4 Faculty of Medicine, University of Freiburg, Freiburg, Germany

**Keywords:** Cavernoma, Spinal AVM, Haemangioblastoma, Ependymoma, Spinal Cord Metastasis, Meningeal Melanoma

## Case Report

**Fig. 1:**
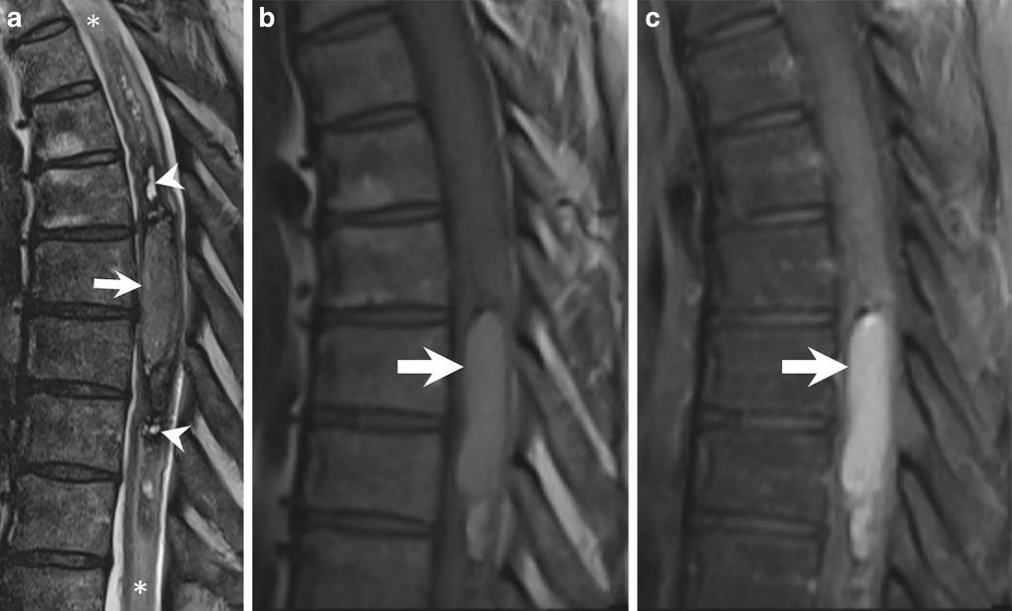
Sagittal T2-weighted MR images **a** show an intramedullary lesion extending from Th6 to Th11. The lesion consists of cystic areas with a hypointense rim (**a**, arrowheads), adjacent to a focal, space-occupying hyperintense portion at the Th8 level (**a**, arrow). Surrounding the lesion, a homogeneous centromedullary hyperintensity indicative of perifocal edema is observed (**a**, asterisks). On T1-weighted images prior to Gadolinium (Gd) administration, the space-occupying portion appears homogeneously hyperintense (**b**, arrow). Following Gd administration, T1-weighted images with fat saturation reveal no contrast enhancement within the lesion (**c**, arrow)

**Fig. 2:**
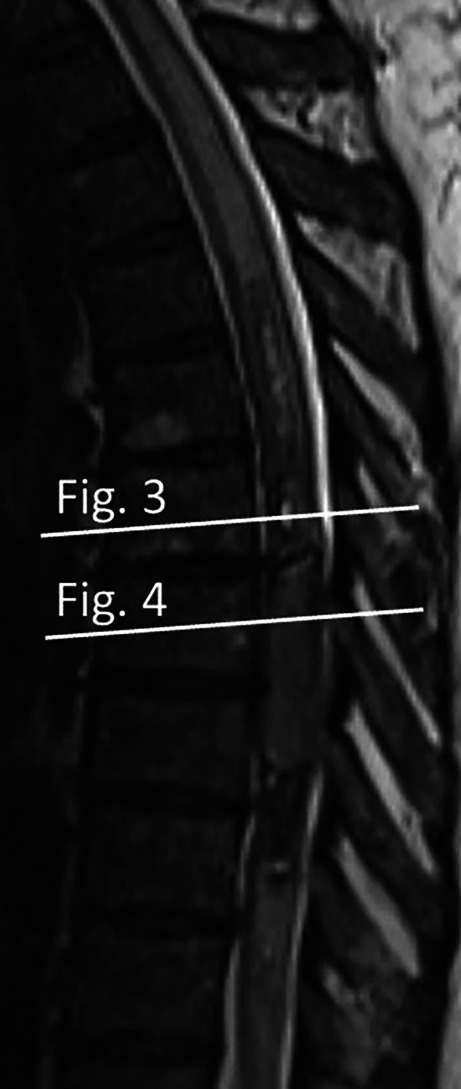
Sagittal T2-weighted MR image serving as a scout for Figs. 3 and 4, illustrating the cross-sectional levels at Th7 (Fig. 3) and Th8 (Fig. 4)

**Fig. 3:**
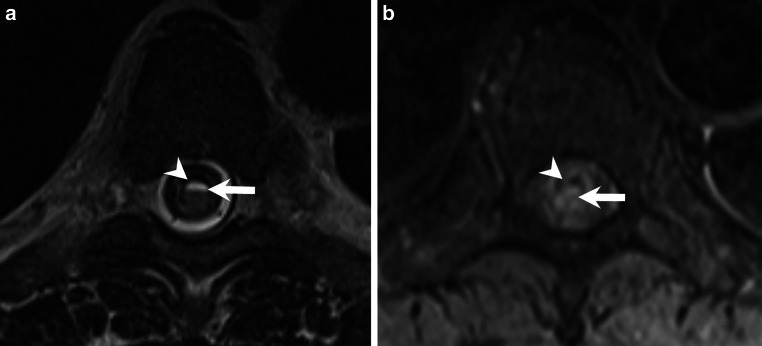
Axial T2-weighted images show the cystic lesions with a fluid-fluid level (**a**, arrow) and a pronounced hypointense rim (**a**, arrowhead). Corresponding axial non-enhanced T1-weighted images at the same level reveal a slightly hyperintense signal relative to the spinal cord (**b**, arrow) and a hyperintense rim (**b**, arrowhead)

**Fig. 4:**
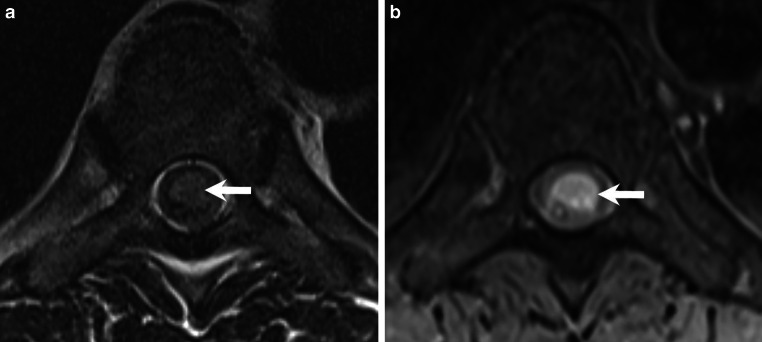
Cross-sectional images at the Th8 level show a slightly hyperintense signal on axial T2-weighted images (**a**, arrow) and a marked hyperintense signal on T1-weighted images without contrast (**b**, arrow) in the space-occupying portion of the lesion

A 57-years-old male patient presented with significant clinical features indicative of a pronounced dorsal column deficit. While he was still able to walk, he exhibited severe gait ataxia, almost complete loss of sensation below Th8 and bladder and fecal incontinence. Magnetic resonance imaging (MRI) of the spine revealed what was initially thought to be an extended intramedullary hematoma (Figs. 1, 2, 3 and 4). Due to the acutely progressive nature of his spinal cord symptoms, decompression of the intramedullary hematoma was recommended.

An en-bloc laminotomy was performed from Th7 to 9 via a midline approach. After opening the dura, a distended myelon with a perforation of a hemorrhagic lesion to the dorsal surface medial to the midline was revealed. A midline myelotomy was then performed from the perforation site. The lesion was then successively developed under continuous monitoring of SEP, MEP and D‑wave. Two components were found: on the one hand, a soft mass most likely corresponding to a haemorrhage. On the other hand, a fibrous tissue matrix suspicious of a tumour. The intraoperative histopathology of the latter suggested a melanoma. Accordingly, a radical resection at the ventral border of the spinal cord was not performed. However, the lesion could be largely removed. Electrophysiologically, an SEP loss, a partial MEP loss to individual muscle groups and an approx. 50% reduction in the amplitude of the D‑wave were noted intraoperatively. Finally, the spinal cord was well decompressed. The dura was then sutured and the laminotomy block repositioned with miniplates. The patient reported an immediate postoperative improvement in leg hypoesthesia. Gait ataxia persisted without any new motor impairment, but he remained able to walk and climb stairs. Over time, he regained continence for both stool and urine.

## Imaging

An MRI performed upon admission revealed an intramedullary lesion extending between the levels Th6 and Th11. The lesion contained cystic areas with a hypointense rim on T2-weighted images (Fig. 1a, arrowheads), adjacent to a focal, space-occupying hyperintense portion at the Th8 level (Fig. 1a, arrow). Surrounding the lesion, a homogeneous centromedullary hyperintensity indicative of perifocal edema was observed (Fig. 1a, asterisks).

On T1-weighted images obtained prior to Gadolinium (Gd) administration, the space-occupying portion appeared homogeneously hyperintense (Fig. 1b, arrow). Following Gd administration, no contrast enhancement was observed within the lesion (Fig. 1c, arrow).

Axial images were obtained at the level of the cystic lesion at Th7 and the space-occupying component at Th8 (scout, Fig. 2). On T2-weighted images, the cystic lesions displayed a fluid-fluid level (Fig. 3a, arrow) and a pronounced hypointense rim (Fig. 3a, arrowhead). A corresponding T1-weighted image at the same level showed a slightly hyperintense signal relative to the spinal cord (Fig. 3b, arrow), as well as a hyperintense rim (Fig. 3b, arrowhead).

Cross-sectional images of the space-occupying lesion at Th8 showed a slightly hyperintense signal on T2-weighted images (Fig. 4a, arrow) and a marked hyperintense signal on T1-weighted images without contrast (Fig. 4b, arrow).

Based on these imaging characteristics, we initially interpreted the lesion as an extensive hematoma of varying ages, with chronic haemorrhages at Th6 and Th9, and a subacute clearly space occupying haemorrhage displaying methemoglobin at Th8. In light of the neuropathological diagnosis, the interpretation of the space-occupying portion of the lesion at the Th8 level should be revisited and can now be regarded as a solid, non-enhancing tumour.

## Differential Diagnosis

Intramedullary hemorrhage is an infrequent finding, and mostly secondary to spinal cord trauma. Non-traumatic intramedullary spinal cord hemorrhage (ISCH) is an even rarer condition, with an estimated incidence of 0.2–0.5 cases per 100,000 individuals annually [1]. The main aetiologies of the non-traumatic intramedullary hemorrhage are arteriovenous malformations and cavernomas, followed by complications of anticoagulant therapy (warfarin), congenital anomalies of coagulation (von Willebrand’s disease, factor VII, VIII or IX deficiency), primary marrow tumours (ependymoma, astrocytoma and primary melanoma of the central nervous system) or metastasis (melanoma, lung, breast and renal cell carcinoma), and less frequently, are secondary to radiotherapy [2, 3]. Key clinical features include sudden onset of severe back pain, neurological deficits such as weakness, sensory loss, or paralysis below the lesion—as observed in our 57-year-old male patient who presented with severe gait ataxia and almost complete loss of sensation below Th8—as well as autonomic dysfunction described in the literature, including bowel or bladder incontinence.

MRI is the gold standard for diagnosing intramedullary spinal cord hemorrhages. Key imaging features include T1-weighted native images with hyperintense signals in the subacute phase and T2-weighted images with mixed hyperintense and hypointense areas due to blood degradation products and edema and associated findings such as spinal cord swelling or compression.

### Spinal Cord Cavernoma

Spinal cord cavernous malformations (SCMs), also known as spinal cavernomas, are rare vascular malformations that account for 12% of all spinal vascular malformations in adults [4] and 1% of intramedullary lesions in children [5] Their prevalence is estimated at 0.4–0.6% in the general population [6], with a peak incidence in the fourth decade of life and a female predominance [7].

SCMs are composed of dilated, low-flow vascular channels lined by endothelium without intervening neural or glial tissue. They are typically located in the thoracic (58%) or cervical (38%) spinal cord. These lesions may remain asymptomatic for long periods [8] but have a high risk of hemorrhage, with an annual bleeding rate of 2–3%, increasing to 9–10% in symptomatic patients or those with prior hemorrhage [9].

Symptoms vary widely, depending on the location and extent of hemorrhage. Acute presentations include sudden back or neck pain, motor deficits, sensory disturbances, and bowel/bladder dysfunction due to intramedullary hemorrhage.

Chronic or progressive symptoms include myelopathy, radicular pain, and episodic neurological decline caused by microhemorrhages or gliosis [10].

MRI typically shows rounded lesions with heterogeneous signal intensity on T1- and T2-weighted images due to blood products of varying ages (“popcorn appearance”) and a hypointense hemosiderin rim on T2-weighted images. Gradient echo sequences reveal hypointense “blooming” due to hemosiderin deposition and minimal cord expansion unless recent hemorrhage has occurred [7, 9] In our patient, the cystic components also exhibited a hypointense rim but lacked the pronounced “popcorn appearance” and blooming in gradient echo sequences which argues against a vascular origin.

### Spinal Cord Arteriovenous Malformation (AVM)

Spinal AVMs are rare, with an incidence of approximately 0.036 per 100,000 person-years [11]. They account for a small percentage of spinal vascular malformations, with hemorrhagic presentations occurring in about 25% of cases [12]. The mean age of diagnosis is around 55–60 years, though symptoms can appear earlier, often in the 20s or even younger in about 20% of cases [13]. Fistulous AVMs are more common in older adults, while nidus AVMs may present at younger ages [12]. Most spinal AVMs are located in the thoracic (54%) or thoracolumbar (32%) regions, with fewer found in the cervical spine (15%) [13].

Symptoms vary based on location and severity and can include sudden severe back pain and segmental neurologic deficits due to hemorrhage [14] Progressive or waxing and waning neurological deficits, including weakness, numbness, tingling, and bowel/bladder dysfunction, radicular pain or gait instability, and in rare cases, subarachnoid hemorrhage may occur with high cervical AVMs [5].

MRI findings include enlarged perimedullar vessels or flow voids visible on T2-weighted images, intramedullary T1 hypointensity and T2 hyperintensity extending over multiple vertebral levels. Hemosiderin deposition from prior hemorrhages may appear as hypointense areas on T2-weighted images [16] In contrast-enhanced MRI may show abnormal vascular structures, but digital subtraction angiography (DSA) remains the gold standard for diagnosis [15]. In our patient, a spinal AVM could be ruled out because neither a nidal structure nor dilated perimedullary veins were detected on imaging.

### Haemangioblastoma

Haemangioblastomas are rare, benign highly vascular tumors of the central nervous system. The overall incidence is approximately 0.141 per 100,000 person-years, with spinal hemangioblastomas accounting for about 2.1% of all intradural spinal tumors [17]. These tumors can occur sporadically or as part of von Hippel-Lindau (VHL) disease. The peak age of diagnosis is between 20 and 50 years, with a lower incidence in children and elderly patients over 65 years [18, 19]. Hemorrhagic events within spinal hemangioblastomas are exceedingly rare but can result in acute and severe neurological deficits. Symptoms may include rapid onset of motor deficits, sensory loss, and bowel/bladder dysfunction as our patient presented with. Haemorrhagic events are particularly rare in cervical hemangioblastomas but have been documented in thoracic lesions [20, 21]. Key MR findings include a hyperintense signal of subacute hemorrhages due to the presence of methemoglobin on T1-weighted images and mixed hyperintense and hypointense areas caused by blood degradation products and surrounding edema on T2-weighted images [20, 22]. Additionally there are prominent flow voids on MRI which indicate the highly vascular nature of the tumor [22]. On T1 post contrast images there is strong enhancement which is typical for hemangioblastomas; however, hemorrhagic regions may show heterogeneous enhancement [23]. In our patient, we observed features suggestive of an intraspinal hemorrhage, yet there were no flow voids or significant contrast enhancement. Furthermore, the patient had no known history of VHL disease, further reducing the likelihood of a hemangioblastoma being the source of the spinal hemorrhage.

### Spinal Cord Ependymoma

Spinal ependymomas are the most common adult intramedullary tumor, accounting for more than 60% of all intramedullary lesions. The peak incidence lays in the 4th decade with a slight male predominance. Prognosis varies by molecular subtype; spinal ependymomas in adults show 5‑year survival rates of 67–85% [24].

The clinical presentation and onset largely depends on the spinal location of the tumor with pain, weakness, and sensory changes common. Both non-specific and specific sensory and/or motor symptoms can be affected [25]. Very large ependymomas can cause motor deficits, as were present in our patient [26]. Ependymomas can occur anywhere along the spinal cord, however, the cervical cord is the most common site (44%). An additional 23% occur within the cervical cord and extend into the upper thoracic cord, and 26% occur in the thoracic cord alone with an average length of four vertebral body segments [27]. The well-circumscribed lesions [25] are strogly homogeneous enhancing and iso- to hypointense on T1 and hyperintense on T2 often with cystic components or syrinx formation.

In our patient, we also identified cystic lesions, and the space-occupying lesion at the Th8 level appeared slightly hyperintense on T2-weighted sequences. On T1-weighted sequences, it showed a hyperintense signal without definite contrast enhancement. Hemorrhagic ependymomas exhibit distinct imaging features in T1-weighted images: areas of hyperintensity due to methemoglobin in subacute hemorrhage phases and in T2-weighted images hypointense margins caused by hemosiderin deposition (cap sign). The MRI characteristics of the lesions in our patient were consistent with the diagnostic features of a hemorrhagic ependymoma.

### Spinal Cord Metastasis

Intramedullary spinal cord metastases (ISCMs) are rare, occurring in 0.9–2.1% of cancer patients based on autopsy studies [28–30]. ISCMs with hemorrhage are even rarer, occurring in approximately 1% of intramedullary spinal cord metastases and most commonly associated with hypervascular or aggressive primary tumors [28–30].

The most frequent cause is lung cancer [31], particularly small cell lung carcinoma, which has a high propensity for leptomeningeal spread and vascular disruption, leading to hemorrhage. Melanoma of the skin is another common culprit due to its aggressive nature and tendency for CNS spreading, including the spinal cord and shows a 6–18% rate of leptomeningeal spread, which can involve fragile neovascularization. Renal cell carcinoma (RCC) is also strongly linked to hemorrhagic metastases because of its hypervascularity, which increases the risk of spontaneous bleeding. Breast cancer, thyroid cancer, and hepatocellular carcinoma (HCC) have been reported as causes of hemorrhagic spinal cord metastases too, often due to their vascular characteristics [29]. These metastases typically present with acute neurological symptoms such as paraparesis or Brown-Séquard syndrome [30]. MRI findings include T1 hyperintensity in the subacute phase and T2 hyperintensity with a hypointense rim in acute hemorrhagic lesions and extensive edema may be present around the lesion. Post-gadolinium enhancement is often intense and can be homogeneous or heterogeneous, which was not present in our patient. However, the MRI features typical of a hemorrhage were evident and also a marked perifocal edema.

In patients over 50 years of age presenting with acute neurological symptoms and a history of malignancy a potential differential diagnosis ISCM must be considered as a differential diagnosis [32]. Definitive confirmation can only be achieved through histopathological analysis.

### Meningeal Melanoma

Meningeal melanoma is a primary melanocytic tumor of the central nervous system (CNS). It accounts for fewer than 1% of all melanoma cases, with only around 50 reported cases worldwide [33]. The tumor typically arises from the leptomeninges. There is no clear gender predilection and the mean age at diagnosis was 54 years (range: 20 to 80 years) according to a publication by Kim et al. reporting on a literature review of 26 cases of meningeal melanomas. The tumors were most commonly located in the thoracic spine (42%) followed by cervical (35%), thoracolumbar (12%), cervicothoracic (8%), and lumbar (5%) manifestations. The clinical symptoms are non-specific, with progressive weakness being the most common presentation. Distinguishing between primary and metastatic melanoma is crucial due to differences in prognosis [33].

The typical MRI signal patterns for primary spinal melanoma include hyperintensity on T1-weighted sequences due to the paramagnetic effects of melanin and hypointensity or isointensity on T2-weighted sequences. Spinal melanomas generally show mild to moderate heterogeneous enhancement on post-contrast sequences [33].

In our case, we observed a space-occupying lesion at the Th8 level that exhibited a homogeneous hyperintense portion on T1-weighted images without conceivable contrast enhancement. Additionally, we noted features of previous hemorrhage and a centromedullary hypersignal, indicating extensive edema. T2 hyperintense areas within the signal-altered spinal cord at the T6–T9 levels were consistent with a subacute hemorrhagic lesion.

Given the rarity of this tumor, there is limited evidence in the literature to reliably define the MRI features of meningeal melanoma. It is possible that meningeal melanomas may present with highly heterogeneous MRI characteristics. In our case, the solid, space-occupying tumor encountered during surgery was histologically confirmed as a meningeal melanoma. However, its MRI characteristics—T1 hyperintensity, slight T2 hyperintensity, and absence of substantial contrast enhancement—deviate significantly from those typically reported in the literature. As a result, we did not initially consider this diagnosis as part of our differential.

It is possible that the MRI characteristics of the menigeal melanoma in our case were altered due to the concurrent hemorrhage within the tumor. Hemorrhagic changes can influence signal intensities on MRI, as previously described, with the paramagnetic effects of melanin and hemorrhagic products, such as methemoglobin, potentially modifying T1 and T2 signal intensities.

## Histology, Immunohistochemistry and Molecular Analyses

**Fig. 5:**
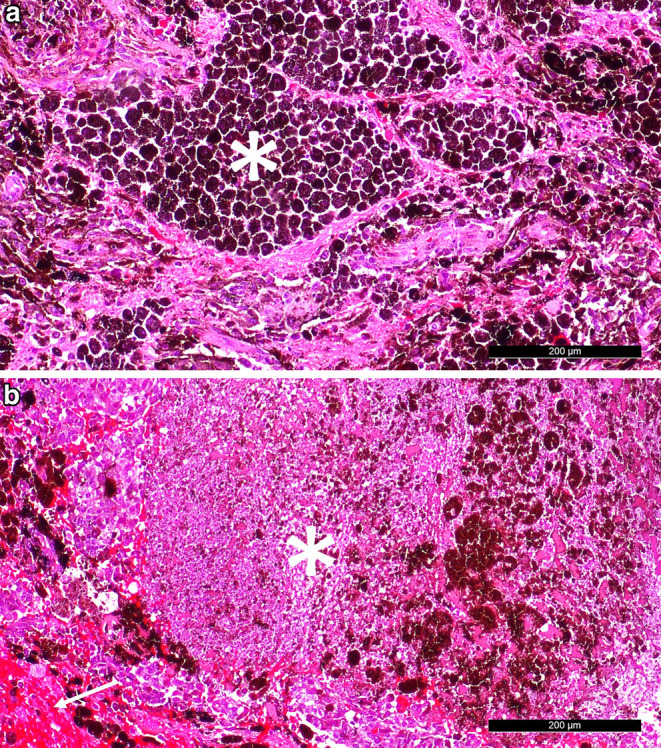
H&E staining reveals **a** a heavily pigmented (indicated with a star) tumour with high cellularity and **b** areas of necrosis as well as signs of fresh haemorrhages. Scale bar = 200 µm

Hematoxylin and Eosin (H&E) staining of the formalin-fixed, paraffin-embedded surgical specimen revealed a pleomorphic tumour with high cellularity, characteristic dark pigmentation and sheath-like growth pattern (Fig. 5a). The majority of the tumour cells contained small, round or oval nuclei and exhibited prominent nucleoli. Further, large areas of fresh haemorrhages could be noted (Fig. 5b) as well as occasional perivascular immune infiltrates. Prussian blue staining showed some iron residues, indicating older haemorrhages as well. While evaluation of proliferation activity was associated the great difficulty given the extensive pigmentation, we observed an increased mitotic activity with 3 mitoses/10 high-power fields (HPF). Further, as a signs of rapid growth some areas of the tumour appeared to be necrotic (Fig. 5b).

**Fig. 6:**
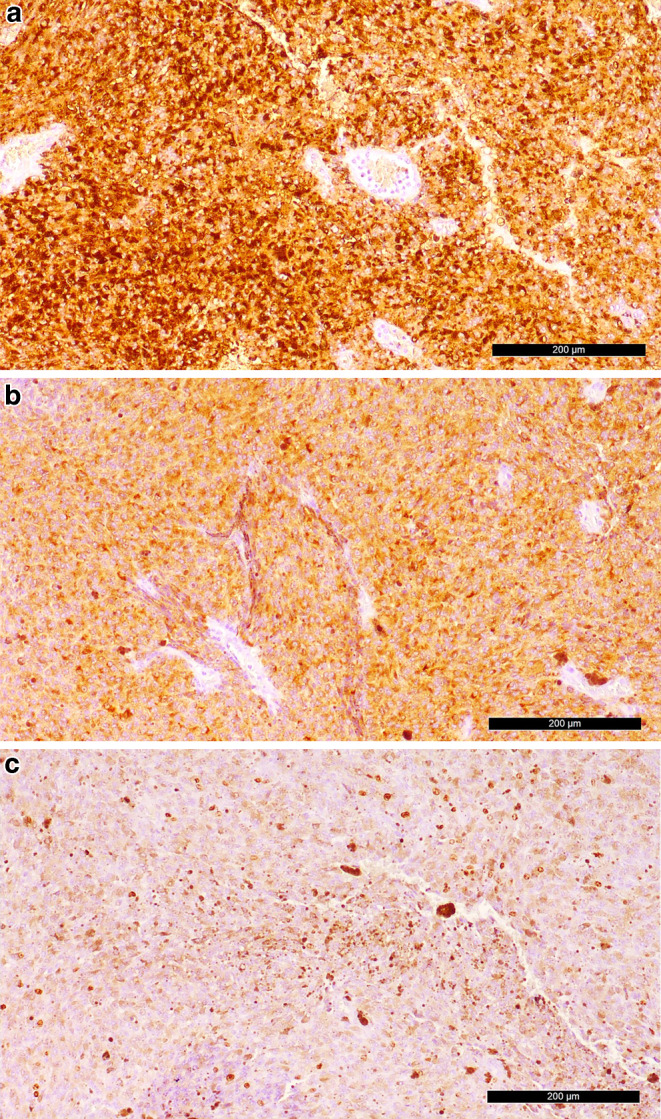
Tumour cells react strongly positive in immunohistochemical analysis of **a** HMB45 and **b** MelanA. **c** MIB‑1 indicates a proliferation rate of 10–15%. Scale bars = 200 µm

Immunohistochemical analysis showed a strong positivity for HMB45, MelanA (Fig. 6a, b) and S100 (not shown) indicating a melanocytic differentiation. The immunohistochemical reaction for MIB‑1 revealed a proliferation rate of 10–15% (Fig. 6c).

**Fig. 7:**
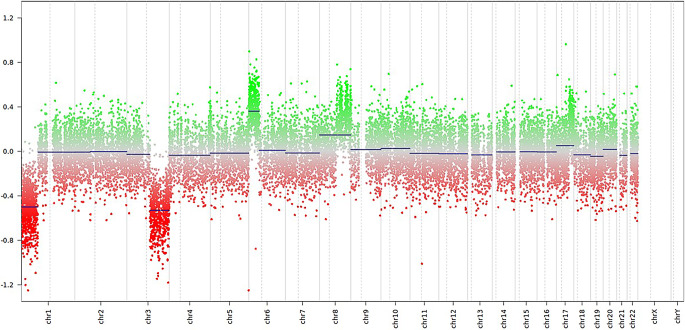
Copy number profiling indicates characteristic losses on chromosome arms 1p and 3q, as well as gains on chromosome arms 6p and 8q

Targeted next-generation sequencing of isolated tumour DNA revealed wildtype sequences for BRAF, KIT, NRAS and GNA11 while showing a gain-of-function mutation of GNAQ (p.Q209L) with a variable allele frequency (VAF) of 41.4% as well as a variant of SF3B1 (p.R625H) with a VAF of 39%. Concurrently, an 850k EPIC array was performed. The inferred copy number variation profile indicated losses on chromosome arms 1p and 3q as well as gains on chromosome arms 6p and 8q (Fig. 7). Using the Heidelberg Epignostix CNS Tumour Classifier v12.8 in which a calibrated score ≥ 0.9 is considered a significant match, the methylation profile of the sample matched the methylation class of “Circumscribed Meningeal Melanocytic Neoplasms” and more specifically the methylation class of “Melanocytoma”, both with a calibrated score of 0.99.

In summary, the morphological pigmentation, the immunohistochemical expression pattern and the molecular findings of a GNAQ mutation as well as a matching methylation profile suggested the diagnosis of a circumscribed meningeal melanocytic neoplasm.

Circumscribed meningeal melanocytic neoplasms are recognized as its own entity in the fifth edition of the WHO classification of central nervous system (CNS) tumours [34]. Based on histopathological features, these tumours are further graded: Meningeal Melanocytomas are well-differentiated and show no signs of anaplasia, such as necrosis, cellular atypia or increased proliferation, as well as no invasion of the CNS parenchyma. Intermediate-grade melanocytoma are histopathologically similar, but can display brisk mitotic activity (1–3 mitoses/10 HBF) or CNS invasion. Meningeal melanomas are characterized by pleomorphic morphology, high cellularity, increased mitotic activity (> 3 mitoses/10 HBF) and often necrosis. The most relevant differential diagnosis are brain metastases of cutaneous malignant melanoma, considering that metastatic melanoma has the highest incidence of leptomeningeal metastasis with 23% [35] and primary meningeal melanomas only account for 0.06–0.1% of all meningeal tumours [36]. Another, rare differential diagnosis is leptomeningeal metastasis of uveal melanoma, which is a primary malignant melanocytic neoplasm arising in the uveal tract, representing 3–5% of all melanoma cases [37]. Therefore, every leptomeningeal lesion suspicious for a malignant melanocytic neoplasm first should be followed up by a thorough dermatological assessment for a potential cutaneous or uveal primary tumour. In absence of such a primary tumour, molecular testing should be initialized. While all of the mentioned entities are indistinguishable based on histopathological features, molecular analysis can help discriminate them: Brain metastasis of malignant melanoma of the skin commonly carry mutations in BRAF, NRAS, KIT or HRAS [38]. These mutations are usually not observed in circumscribed meningeal melanocytic neoplasms. By contrast, typical molecular alterations of these tumours include gain-of-function mutations in GNAQ, GNA11, PLCB4 or CYSLTR2 as well as chromosomal alterations including gains on chromosome arms 8q and 6p as well as losses on chromosome arms 1p and 6q [39]. Uveal melanoma metastases are characterized by the same hallmark genetic alterations as meningeal melanocytic neoplasms, making it very difficult to distinguish them on a molecular level [37]. This highlights the necessity of an interdisciplinary clinical approach for the diagnostic process of melanocytic tumours located in the leptomeninges.

### Diagnosis: Meningeal Melanoma

Molecular profiling revealed a GNAQ mutation as well as chromosomal gains on 8q and chromosomal losses on 1p, all considered genetic hallmarks of circumscribed meningeal melanocytic neoplasms. In line with the histopathological findings of increased mitotic activity, pleomorphic morphology and necrosis, we identified a SF3B1 genetic variant that is associated with aggressive clinical behaviour [40]. Therefore, we considered this tumour to be a meningeal melanoma. This entity is considered extremely rare and has an estimated annual incidence of 0.005 cases per 100,000; however, this may be an underestimation due to under-diagnosis [39, 41].

Biologically, these tumours originate from leptomeningeal melanocytes. Small numbers of their precursors, so-called melanoblasts, populate the leptomeninges during embryonic development [39]. Meningeal melanomas most frequently occur in the spinal cord and the posterior fossa, but can arise along the whole neuroaxis [34]. They rarely spread extracranially, so evidence of systemic disease would be highly suggestive of metastatic cutaneous or uveal melanoma [39].

Given the lack of large clinical studies, prognostic information and recommendations regarding clinical management are based on small case series. While in a large case series the most favourable outcome of a mean survival of 19.58 months was achieved with gross total resection [41], there is less agreement on post-operative treatment options and different combinations of radiotherapy and intrathecal or systemic chemotherapy have been proposed [42]. Anecdotally, in the presented case the patient received postoperative focal radiotherapy as well as the immune checkpoint inhibitor Nivolumab, which has been associated with a long-lasting therapeutic response in a recent case report [43].
